# Post-operative pain management using two drugs following dental implant surgery among Indians

**DOI:** 10.6026/97320630019476

**Published:** 2023-04-30

**Authors:** Surendran Sundaram, Sahana Selvaganesh, Thiyaneswaran Nesappan, Vishnu Priya Veeraraghavan, Rajalakshmanan Eswaramoorthy

**Affiliations:** 1 Department of Implantology, Saveetha Dental College and Hospitals, Saveetha Institute of Medical and Technical Sciences, Saveetha University, Chennai 600077, India; 2 Department of Biochemistry Saveetha Dental College and Hospitals, Saveetha Institute of Medical and Technical Sciences, Saveetha University, Chennai 600077, India; 3 Department of Biomaterials, Centre of Molecular Medicine and Diagnostics (COMManD), Saveetha Dental College and Hospitals, Saveetha Institute of Medical and Medical and Technical Sciences, Saveetha University, Chennai 600077, India

**Keywords:** Pain management, acetaminophen, trypsin, chymotrypsin, dental implant

## Abstract

It is of interest to assess two drug regimens for managing pain and swelling in 40 dental implant surgery patients. Visual analogue scale readings were taken at 24 hours, 72 hours and 1 week. Data shows that a combination of acetaminophen and aceclofenac with trypsin - chymotrypsin was found to be more effective than acetaminophen alone.

## Background:

Dental implant has become the treatment of choice to manage complete and partially edentulous situations, the treatment has undergone huge transformation to improve the accuracy of the therapy, but the surgical procedure still remains the same, which requires osteotomy of the implant site, which could cause considerable discomfort to the patient. It is a known fact that people tend to be anxious towards dental therapy, [1] epidemiological study of the South Indian population reported that 51.8% patients were moderately or extremely anxious and 3% were suffering from dental phobia, 82.6% of patients were averse to extraction procedures, in such situation the patient tends to avoid treatment. It has also been reported [2] that overall incidence of postoperative pain was 40.4%, the incidence and severity of the occurrence of pain depended upon the procedure the highest being pain perceived after root canal therapy and lowest after restorations, there also reports on gender related variations on pain perception, with females experiencing it more compared to males. In various clinical studies it has been observed that a combination of NSAIDs proved to be beneficial in controlling pain at any point postoperatively compared to a single dose of paracetamol [3][4][5]. A combination of different analgesics may produce the intended effect with fewer side effects when compared to a single drug and the current trend is the use of such combinations in management of acute pain, but the clinical superiority of such combination of paracetamol and an NSAID over either drug alone remains controversial [6]. Therefore, it is of interest to understand the efficiency of acetaminophen as a standalone analgesic intervention compared to aceclofenac combined with trypsin - chymotrypsin.

## Materials and Methods:

## Selection of Subjects:

Approval of the study protocol and ethical clearance were obtained from the Institutional Review Board, Saveetha Dental College Hospitals, Chennai, India and were within the statutory limitations of the Revised Helsinki Declaration of World Health Organization 2013. The study design as described elsewhere [7, 8, 9, 10, 11, 12, 13].

An informed consent was also obtained from the patients who were enrolled in the study. This prospective, single blinded clinical pilot study was done at the Department of Implantology, Saveetha Dental College and Hospital.

##  Sample Size:

The sample was calculated considering the mean expected difference and pooled standard deviation obtained from previous literature. The minimum sample size for this pilot study having two groups required to observe the difference (with type I error 5% and power at 80%) was 20 participants per group to account for the potential refusal to participate, or loss of patient during trial.

## Inclusion and Exclusion criteria:

The inclusion criteria included individuals undergoing dental implant surgery where a single root form endosseous implant was placed in either jaw without immediate loading or restoration done, patients with notable systemic ailments such as uncontrolled diabetes, immunological disorder, osteoporosis were excluded from the study.

## Subject allocation and randomization method:

The patients were allocated based on random selection of the outpatient number in the two groups, they were given a sealed envelope with their corresponding group ID, the intervention to be given was not relieved to the patient, and the drugs were given in unlabelled pouches.

## Intervention:

After obtaining the informed consent for the surgical procedure, local anaesthesia - nerve block for mandibular site or local infiltration for the maxillary site administered using 1 to 2 doses of (1.8 mL each) of anaesthetic solution (2% lidocaine with 1:80,000 adrenaline, Xicaine; ICPA [ICPA Health Products Ltd. Mumbai]), incision and full thickness periosteal flap raised osteotomy sequence initiated, orientation of the same verified with the position indicating device and radiographs, the preparation is then completed and implant (Nobel PMC Replace Select; Nobel Biocare [Switzerland]) inserted and a primary stability of 35 NCm obtained, the displaced tissues were then sutured back using 4-0 polyamide sutures (ORLON monofilament polyamide ; ORION Sutures [Orion Sutures India Pvt Ltd. Bangalore]) post-operative instructions were given and the drugs (Acetaminophen 650 mg, Tablet Dolo 650; [Micro Labs Ltd. Bangalore] and Aceclofenac 100 mg combined acetaminophen 325 mg and trypsin chymotrypsin 50,000 AU, Tablet Chymoral - AP; [Torrent Pharmaceuticals Ltd. Ahmedabad]) under investigation were given to patients in unlabelled pouches and they were requested to take the same twice daily orally, the patients were called back after 24 hours, 72 hours and after 1 week.

## Assessment of pain after dental implant surgery:

Patients pain intensity experience was measured using visual analog scale (VAS), which has a 10 cm line anchoring the two extreme outcome parameters, one being "no pain" and the other end marked as "pain as bad as it could be". The patient was asked to mark relevant answer on the given sheet having the VAS scale.

## Statistical analysis:

Normality of the data was tested using Shapiro - Wilks test, because the data showed normal distribution, parametric tests - paired sample t test was done and Cohen D was used to calculate the size of the effect at the different time intervals, the data was analysed using IBM SPSS Statistical Software version 23.

## Results:

A total of 40 patients participated in this study, out of which 20 patients were male and the rest were females. The average age of the participants was 34.5 ± 11.5 years, there was no attrition of the sample and follow-up was done for all participants. Paired sample t test was done, and it was observed that the administration of the combined drug had a statistically significant effect at 24 hours and 72 hours' time interval, the values tabulated in Table1. On further exploration of the effect size it was observed that group 1 had a higher mean compared to group 2 at all three time points, with decreasing effect sizes over time, that is the largest effect size at 24 hours (d =5.66), medium effect size at 72 (d = 1.96) hours and small effect size at 1 week (d = 0.4) as demonstrated in Table2. Thus, the variation in values was considered to be statistically significant. On evaluation it is observed from the mean and SD values; group2 had better outcome compared with group1 and there is statistically significant difference in pain perception at the 24 hours and 72 hours interval compared to one week.

## Discussion:

Careful selection of an effective analgesic regimen should be based on the type and quantum of pain the patient is expected to perceive, and it is important to develop safer and effective analgesic regimens. Various clinical trial reports on diclofenac combined with trypsin - chymotrypsin is said to be more effective compared to plain diclofenac [14], post third molar extraction acetaminophen ibuprofen combination had no pharmacokinetic interaction and the pain management potential was greater when the drugs were combined [15]. It has been reported that the site of action of acetaminophen therefore the analgesic effect is considered synergistic when combined with NSAIDs [6], the review by Bailey et al. where it was concluded that pain intensity in the combination group was effective had lesser when compared to single analgesic such as acetaminophen and the need for rescue medication is reduced, patients were observed to have reduced pain experience for a period of 6- 8 hours after administration of combination of NSAID and aceclofenac. Chymotrypsin is a type of serine protease; it uses a serine residue to catalyse the hydrolysis of peptide bonds in proteins. It specifically targets peptide bonds adjacent to aromatic amino acids, such as tyrosine, phenylalanine, and tryptophan [16]. It targets early stage of inflammation, reducing the period of fibrinolysis, as a result local microcirculation is improved reducing the extent of edema, inflammation aiding faster recovery and healing, this compound is also having analgesic but when compared to diclofenac has lesser analgesic potential. This drug is a proteolytic enzyme, and it has been observed to have an analgesic property close to that of NASIDs [17] Based on the outcomes of the current study it can be understood that a combination of NSAID and the proteolytic enzyme offers better postoperative analgesia compared to that of a single dose of acetaminophen, it was noted that the administration of the combination effectively reduce the postoperative pain sequelae at the 24 hour level waning in action and offered similar results at the end of 1 week to the stand alone drug, and the patients had an optimistic response to the drug combination, this study thus can be the basis for further investigations on wider patient population would help to add more significance to this finding.

## Conclusion:

Data shows that a combined use of aceclofenac with acetaminophen trypsin - chymotrypsin has more sustained effect in reducing post dental implant surgery compared to stand alone analgesic among Indians.

## Figures and Tables

**Table 1:** VAS Score Means, SD and t - distribution of the two intervention groups.

**Time interval**	**Mean ± SD of VAS Score**		**t value**	p*
	Group 1 (n = 20)	Group 2 (n = 20)		
24 hours	7.5 ± 1.20	1.5 ± 0.92	10.39	.000*
72 hours	4.6 ± 1.5	1.9 ± 1.2	3.94	.003*
1 week	0.5 ± 0.52	0.3 ± 0.48	1	0.343
+ Group 1 - Acetaminophen; ++ Group 2 - Aceclofenac/acetaminophen/trypsin-chymotrypsin; *Significant p<0.005

**Table 2:** Effect size calculation using Cohen D

	**24 hours**	**72 hours**	**1 week**
Pooled Standard Deviation	1.06	1.36	0.5
Cohen's d	5.66	1.96	0.4
From this test it can be concluded that the administration of the drug has a considerable effect at 24 hours; 72 hours and gradually reduces by 1 week.
